# Comprehensive Assessment of *BARD1* Messenger Ribonucleic Acid Splicing With Implications for Variant Classification

**DOI:** 10.3389/fgene.2019.01139

**Published:** 2019-11-19

**Authors:** Logan C. Walker, Vanessa Lilian Lattimore, Anders Kvist, Petra Kleiblova, Petra Zemankova, Lucy de Jong, George A. R. Wiggins, Christopher Hakkaart, Simone L. Cree, Raquel Behar, Claude Houdayer, kConFab Investigators, Michael T. Parsons, Martin A. Kennedy, Amanda B. Spurdle, Miguel de la Hoya

**Affiliations:** ^1^Department of Pathology and Biomedical Science, University of Otago, Christchurch, New Zealand; ^2^Division of Oncology and Pathology, Department of Clinical Sciences Lund, Lund University, Lund, Sweden; ^3^Institute of Biology and Medical Genetics, First Faculty of Medicine, Charles University and General University Hospital in Prague, Prague, Czechia; ^4^Institute of Biochemistry and Experimental Oncology, First Faculty of Medicine, Charles University, Prague, Czechia; ^5^Molecular Oncology Laboratory, CIBERONC, Hospital Clinico San Carlos, IdISSC (Instituto de Investigación Sanitaria del Hospital Clínico San Carlos), Madrid, Spain; ^6^Department of Genetics, F76000 and Normandy University, UNIROUEN, Inserm U1245, Normandy Centre for Genomic and Personalized Medicine, Rouen University Hospital, Rouen, France; ^7^Sir Peter MacCallum Department of Oncology, University of Melbourne, Melbourne, VIC, Australia; ^8^Research Department, Peter MacCallum Cancer Center, Melbourne, VIC, Australia; ^9^Department of Genetics and Computational Biology, QIMR Berghofer Medical Research Institute, Brisbane, QLD, Australia

**Keywords:** breast cancer, mRNA splicing, nanopore sequencing, RNAseq analysis, variant classification, ACMG

## Abstract

**Introduction:** Case–control analyses have shown *BARD1* variants to be associated with up to >2-fold increase in risk of breast cancer, and potentially greater risk of triple negative breast cancer. *BARD1* is included in several gene sequencing panels currently marketed for the prediction of risk of cancer, however there are no gene-specific guidelines for the classification of *BARD1* variants. We present the most comprehensive assessment of *BARD1* messenger RNA splicing, and demonstrate the application of these data for the classification of truncating and splice site variants according to American College of Medical Genetics and Genomics and the Association for Molecular Pathology (ACMG/AMP) guidelines.

**Methods:** Nanopore sequencing, short-read RNA-seq (whole transcriptome and targeted), and capillary electrophoresis analysis were performed by four laboratories to investigate alternative *BARD1* splicing in blood, breast, and fimbriae/ovary related specimens from non-cancer affected tissues. Splicing data were also collated from published studies of nine different tissues. The impact of the findings for PVS1 annotation was assessed for truncating and splice site variants.

**Results:** We identified 62 naturally occurring alternative spliced *BARD1* splicing events, including 19 novel events found by next generation sequencing and/or reverse transcription PCR analysis performed for this study. Quantitative analysis showed that naturally occurring splicing events causing loss of clinically relevant domains or nonsense mediated decay can constitute up to 11.9% of overlapping natural junctions, suggesting that aberrant splicing can be tolerated up to this level. Nanopore sequencing of whole *BARD1* transcripts characterized 16 alternative isoforms from healthy controls, revealing that the most complex transcripts combined only two alternative splicing events. Bioinformatic analysis of ClinVar submitted variants at or near *BARD1* splice sites suggest that all consensus splice site variants in *BARD1* should be considered likely pathogenic, with the possible exception of variants at the donor site of exon 5.

**Conclusions:** No *BARD1* candidate rescue transcripts were identified in this study, indicating that all premature translation-termination codons variants can be annotated as PVS1. Furthermore, our analysis suggests that all donor and acceptor (IVS+/−1,2) variants can be considered PVS1 or PVS1_strong, with the exception of variants targeting the exon 5 donor site, that we recommend considering as PVS1_moderate.

## Introduction

The *BARD1* gene (MIM# 601593) was identified in 1996 as the result of a yeast two-hybrid screen for proteins that interact with the breast and ovarian cancer associated BRCA1 protein (Wu et al., 1996). The *BARD1* reference transcript contains 11 exons and produces a full length 777 amino acid protein which is structurally related to BRCA1 as both contain N-terminal RING finger domains and two carboxy-terminal (BRCT) domains (Miki et al., 1994; Wu et al., 1996). The interaction of BARD1 to BRCA1 is mediated by their respective RING domains leading to the proposal that *BARD1* is a candidate breast and ovarian cancer predisposing gene. Various lines of evidence suggest BARD1 may act as a potent tumor suppressor, including the ability to induce TP53-dependent apoptosis (Irminger-Finger et al., 2001), and the observation that homozygous loss of *BARD1* in mice is embryonically lethal, mimicking the properties of *BRCA1* (McCarthy et al., 2003). Furthermore, numerous studies of individuals who have a family history of breast cancer have found rare and functionally deleterious variants in *BARD1* (Ishitobi et al., 2003; Karppinen et al., 2004; De Brakeleer et al., 2010; Ratajska et al., 2012). Case–control analyses have shown *BARD1* loss of function variants to be associated with a low (< 2-fold) to moderate (> 2-fold) increase in risk of breast cancer (Couch et al., 2017; Kurian et al., 2017; Slavin et al., 2017) and up to five-fold increase in risk of triple negative breast cancer (Shimelis et al., 2018). However, the utility of *BARD1* sequencing to identify actionable pathogenic variants in a clinical setting remains undefined and requires a thorough investigation of all possible ways a variant might lead to loss of function. Sequence variants play an important role in the regulation of pre-messenger RNA (mRNA) splicing (Scotti and Swanson, 2016), and there is an established link between aberrant splicing of cancer predisposition genes and breast cancer risk (Walker et al., 2010; Whiley et al., 2010; Whiley et al., 2011). Thus, investigating the role of *BARD1* variants in the production of aberrant mRNA transcripts can be used to assess the likelihood of sequence variants causing functional changes that confer pathogenicity (Walker et al., 2013).

Determining the effect of sequence variants on the expression of mRNA splice isoforms and interpreting which spliceogenic variants are potentially deleterious is a major challenge. Reverse transcriptase-polymerase chain reaction (RT-PCR) has been the major technology used to assess mRNA splicing in a variety of cancer susceptibility genes, including *BARD1*. However, incorrect positioning of PCR primers can result in key splicing events not being detected and lead to a misinterpretation of splicing events. For example, *BRCA1* c.594−2A > C was originally classed as pathogenic and associated with an aberrant mRNA profile that included exon 10 skipping (out-of-frame) but no consideration was given to natural alternative splicing (Tesoriero et al., 2005). More recently, we showed that *BRCA1* c.594−2A > C occurs in *cis* with *BRCA1* c.641A > G and should not be considered as a high-risk pathogenic variant because the out-of-frame splicing alteration did not affect the predominant alternative spliced event, Δ(E9_E10), which retains tumor suppressor activity (de la Hoya et al., 2016).

Massively parallel complementary DNA sequencing (RNA-seq) has further advanced our ability to characterize and quantify gene transcripts, and will therefore become a key technology for measuring gene expression changes in clinical diagnostics. Recent studies have begun to demonstrate the utility of RNA-seq for identifying mRNA splicing events in breast cancer susceptibility genes, including *BRCA1* (Davy et al., 2017; de Jong et al., 2017; Hojny et al., 2017), *BRCA2* (Davy et al., 2017), *PALB2* (Lopez-Perolio et al., 2019), and *BARD1* (Davy et al., 2017). These studies revealed that key advantages of using RNA-seq over RT-PCR is the ability to quantitatively assess multiple splicing events across the whole transcript in one sequencing assay. Furthermore, long-read nanopore sequencing is capable of generating sequences of full-length transcripts and thus can resolve complex exon structures of full-length mRNAs from genes expressing a large number of isoforms (de Jong et al., 2017). Several reports have profiled *BARD1* transcripts to characterize “naturally occurring” mRNA splice isoforms across multiple tissue types (Li et al., 2007; Lombardi et al., 2007; Sporn et al., 2011; Bosse et al., 2012; Zhang et al., 2012; Pilyugin and Irminger-Finger, 2014; Davy et al., 2017). However, despite previous published reports of *BARD1* splicing, current catalogues of alternatively spliced events (e.g., Ensembl—ENSG00000138376) only account for a fraction of transcripts associated with this gene.

We present the most comprehensive assessment of *BARD1* mRNA splicing generated by both RT-PCR and RNA-seq (long-read and short-read) platforms across multiple tissue types. Furthermore, we also utilize American College of Medical Genetics and Genomics and the Association for Molecular Pathology (ACMG/AMP) guidelines (Richards et al., 2015), bioinformatic splicing, and population frequency data to evaluate potential pathogenicity of *BARD1* variants located at canonical splice sites. Results from our study provide an important basis to standardize the clinical classification and reporting of *BARD1* genetic variants.

## Materials and Methods

### Ribonucleic Acid Samples

RNA samples assessed in this study were isolated from different tissue types, including 47 human lymphoblastoid cell lines (LCLs) derived from female healthy controls, an epithelial enriched area of nine healthy breast samples from women with breast tumors (SCAN-B study, ClinicalTrials.gov identifier: NCT02306096), two normal fimbria tissues obtained from prophylactic oophorectomies performed in post-menopausal women without cancer, commercially available RNA from one non-malignant breast tissue (Clontech 636576), and one pool of three non-malignant ovarian tissues (Clontech 636555) (**Supplementary Figure S1**).

### Nanopore-Sequencing—MinION Platform

#### Laboratory 1

The Oxford Nanopore MinION Genomic DNA sequencing of LCL RNA was carried out as previously described (de Jong et al., 2017). Briefly, PCR products were prepared for sequencing using the Nanopore Sequencing Kit SQK-NSK007 (R9 Version). Primer sequences for *BARD1* exons 1 and 11 are as follows: 5’-CTCGACCGCCTGGAGAAG-3’ and 5’-CTGGCTTGGGCTTTCTACTG-3.’ The raw electrical signal was uploaded to Metrichor (version 1.107), using the 2D Basecalling RNN for SQK-NSK007. Full-length alternative isoform analysis of RNA (FLAIR; https://github.com/BrooksLabUCSC/flair) was used to identify novel and known isoforms of *BARD1*. Sequence reads in FASTA format were aligned to the GRCh38 using the align module, which implements minimap2 with the splice option. Aligned reads were then corrected and collapsed using the respective modules of FLAIR with default settings. Annotation for known isoforms were provided by GENCODE (v29).

### Targeted Ribonucleic Acid Sequencing—Illumina Platform

#### Laboratory 2

RNA-sequencing of a 36 LCLs from kConFab [18 sample pairs with/without nonsense-mediated mRNA decay (NMD) inhibition] was carried out using Kapa RNA HyperPrep Kit (Roche) according to manufacturer. Briefly, 250 ng of total RNA were chemically fragmented (mean fragment length 200 bp). PCR amplifications were run for 8 and 12 cycles for pre- and post-hybridization PCR, respectively. Plexes of six barcoded samples (166 ng of each) were hybridized with custom-designed SeqCap EZ Choice CZECANCA v1.2, Roche (Soukupova et al., 2018). Libraries were paired-end sequenced on NextSeq 500 with NextSeq 500/550 Mid Output Kit v2.5 (150 cycles). Splice junctions were included if they were identified in at least three LCLs with an average of more than four reads per LCL.

### Whole Ribonucleic Acid sequencing—Illumina Platform

#### Laboratory 1

RNA-sequencing of a single LCL from a female healthy control was carried out as described previously (Lattimore et al., 2018). Briefly, libraries were prepared from total RNA using poly(A) enrichment of the mRNA (mRNA-Seq) to remove ribosomal RNA (rRNA). The calculation of the percentage of junction reads was carried out as described previously (Davy et al., 2017).

#### Laboratory 3

RNA-sequencing of normal breast and fimbria tissue was carried out as previously described [(23) **Supplemental Material** section 1.2 therein]. Briefly, fresh breast tissue was preserved in RNAlater (Ambion) and fimbriae tissue was fresh frozen. RNA was extracted using AllPrep (Qiagen) and libraries prepared with a modified version of the dUTP (Deoxyuridine Triphosphate) method (breast samples) or the TruSeq Stranded mRNA Library Prep Kit (fimbriae samples, Illumina, San Diego, CA). Libraries were paired-end sequenced on an Illumina HiSeq 2000 (2x50 bp, two breast samples) or a NextSeq 500 (2x75 bp, remaining seven samples). Sequence reads were analyzed as described previously (Lopez-Perolio et al., 2019).

### Sequencing Data Availability

The raw data supporting the conclusions of this manuscript will be made available by the authors, without undue reservation, to any qualified researcher.

### Reverse Transcription Polymerase Chain Reaction Assays

#### Laboratory 4

RT-PCR analysis was carried out on 10 LCLs, breast tissue (Clontech 636576), and one pool of three non-malignant ovarian tissues (Clontech 636555) as previously described (Lopez-Perolio et al., 2019). Primer sequence and details as shown in **Supplementary Table S1**.

### Annotation of Alternative Splicing Events

**Figure 1:**
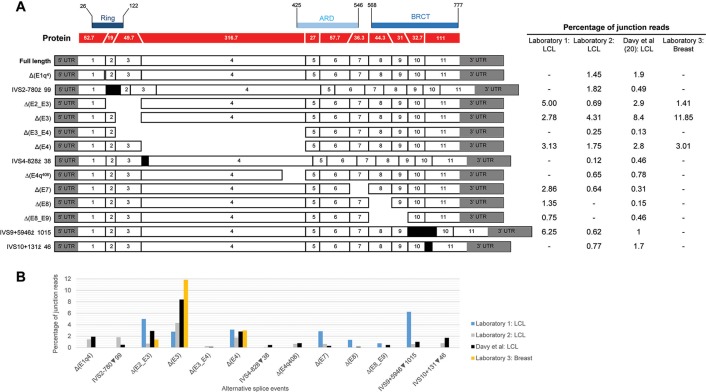
Quantitative expression of *BARD1* splicing events. **(A)** BARD1 protein is shown in red with number of codons per exon (parallel lines represent in-frame junctions) and the location of known functional domains (RING, ARD, and BCRT) are indicated. Schematics of full length and alternative *BARD1* transcripts (white and gray) detected by at least two RNA-seq studies from contributing laboratories 1–3 and published study Davy et al. (2017) are assembled along with the percentage of junction reads found by each laboratory. **(B)** The percentage junction reads associated with splicing events found across the four laboratories.

Alternative splicing events were annotated according to the Human Genome Variation Society (HGVS) guidelines, using the Ensembl transcript ENST00000260947.8 (NCBI RefSeq NM_000465.3) as a reference. Splicing events were also coded as described previously (Lopez-Perolio et al., 2019) using the following symbols: Δ (skipping of reference exonic sequences), ▼ (inclusion of reference intronic sequences), E (exon), I (intron), p (acceptor shift), q (donor shift), and int (interstitial deletion within an exon). Where possible, the exact number of nucleotides skipped (or retained) is indicated. All *BARD1* alternative splicing events reported are predicted to alter the encoded protein. To decide if the truncated/altered region is critical to protein function, we considered the RING, ARD (ankyrin repeat domain), and BRCT domains as shown in **Supplementary Table S2** and **Figure 1**.

### Classification of Splice Site Variants Using American College of Medical Genetics and Genomics and the Association for Molecular Pathology Guidelines

Adaptation of the ACMG/AMP PVS1 decision tree (Abou Tayoun et al., 2018) to *BARD1* donor and acceptor “consensus” dinucleotide (IVS+/− 1,2) variants is detailed in the **Supplementary Methods**.

## Results and Discussion

### *BARD1* Isoform Discovery and Annotation

We present a comprehensive *BARD1* mRNA splicing catalogue from splicing assays of 12 tissue types (normal and cancer tissue) derived from this study and seven publications (**Table 1**). Targeted and whole RNA-seq performed by contributing laboratories produced 299,479 reads aligned to exon-exon junctions at the *BARD1* locus. Targeted RNA-seq of the 36 LCLs by laboratory 2 yielded 292,143 *BARD1* junction reads, whole RNA-Seq yielded 6,656 junction reads of a single LCL (laboratory 1), and 573 and 107 junction reads respectively from 9 breast and 2 fimbria samples (laboratory 3). A total of 62 alternative *BARD1* splicing events were identified in this study. Of these, 19 novel splicing events were found in this study by four contributing laboratories using nanopore sequencing, short-read RNA-seq, and/or RT-PCR. The most commonly found alternative splicing event across studies was the out-of-frame Δ(E4), identified by all technologies in all but one of the tissues assayed. Furthermore, skipping events that included exon 4 were observed in 28 isoforms, suggesting that the absence of this 950 nucleotide exon in a small fraction (up to 3%; **Figure 1**) of *BARD1* transcripts is tolerated by different cell types. We observed no *BARD1* splicing events that were expressed exclusively in breast and/or ovarian tissue. LCLs have been a common cell type used for *in vitro* assays assessing splicing changes in patients with potential spliceogenic variants. Our data showed that there were 12 splicing events [(IVS1+4279▼98, Δ(E2q), Δ(E2_E7), Δ(E3_E4,E7), Δ(E4q137), Δ(E4int104), Δ(E5), Δ(E5_E9), IVS6+4684▼67, ▼(I7q4), IVS9-6318▼92, IVS9+5946▼1015) specific to LCLs. Three of these events were detected exclusively by nanopore sequencing (Δ(E2_E7), Δ(E3_E4,E7), Δ(E5)] and eight were detected by short-read RNA-seq which was used for more LCL samples and with a greater depth of coverage (higher number of junction reads) than for any other tissue type (**Table 1**).

**Table 1:** List of *BARD1* isoforms across 12 tissue types.

Splicing event description	Transcript structure	Functional annotation	LCL					PBL	Breast		Breast tumor	Fimbria	Ovary	Cell line	Fetal sympathetic ganglia	Neuroblastoma	HeLa cells	Colon cancer		Trophoblasts
			NS	RS	TRS	RP	TRS	RP	RS	RP	RP	RS	RP	RP	RP	RP	RP	RP	RP	RP
			Laboratory 1 (n=1)	Laboratory 1 (n=1)	Laboratory 2 (n=36)	Laboratory 4 (n=10)	Davy et al., 2017	Lombardi et al., 2007	Laboratory 3 (n=9)	Laboratory 4 (n=1)	Lombardi et al., 2007	Laboratory 3 (n=2)	Laboratory 4 (n=3)	Pilyugin and Irminger-Finger, 2014	Bosse et al., 2012	Bosse et al., 2012	Li et al., 2007	Sporn et al., 2011	Zhang et al., 2012	Li et al., 2007
Full length	-	IF	+	N	N	+	N	+	N		+	N		+	+	+	+	+	+	+
Δ(E1_E4p)	r.155_?del	?												+						
Δ(E1q4)	r.155_158del	FS		+	+	+	+		+	+			+		+					
Δ(E1q4,E4)	r.155_1314del	IF		N	N		N		N			N			+					
IVS1+4279▼98	r.158_159ins158+4279_158+4376	FS		+	+		+													
Δ(E2)	r.159_215del	IF			+												+	+		
Δ(E2q)	r.212_215del	FS			+		+													
Δ(E2_E3)	r.159_364del	FS		+	+	+	+		+	+		+	+	+	+	+	+		+	+
**Δ(E2,E4-E9)**	**r.159_215+365_1903del**	**IF**		**N**	**N**	**+**	**N**		**N**	**+**		**N**	**+**							
Δ(E2_E4)	r.159_1314del	FS	+	+	+	+		+		+	+				+	+		+		
Δ(E2_E4,E7)	r.159_1314+1569_1677del	FS		N	N		N		N			N						+		
Δ(E2_E4, E8)	r.159_1314+1678_1810del	FS	+	N	N		N		N			N						+		
Δ(E2_E4,E8_E9)	r.159_1314+1678_1903del	FS		N	N		N		N			N						+		
Δ(E2_E4,E8p)	r.159_1314+1678_?del	?		N	N		N		N			N			+					
Δ(E2_E5)	r.159_1395del	FS													+					
Δ(E2_E6)	r.159_1568del	IF	+		+			+			+			+	+	+	+	+	+	+
Δ(E2_E6,E8)	r.159_1568+1678_1810del	FS		N	N		N	+	N		+	N								
Δ(E2_E6,E8_E9)	r.159_1568+1678_1903del	FS		N	N	+	N		N			N			+					
**Δ(E2_E7)**	**r.159_1677del**	**FS**	**+**																	
Δ(E2_E9)	r.159_1903del	FS	+		+	+	+	+		+	+		+	+	+	+	+	+	+	+
IVS2-780▼99	r.215_216ins216-780_216-682	IF		+	+	+	+			+			+							
Δ(E3)	r.216_364del	FS	+	+	+	+	+		+	+		+	+			+		+	+	
Δ(E3_E4)	r.216_1314del	FS	+	+	+	+	+	+		+	+		+					+		
**Δ(E3_E4,E7)**	**r.216_1314+1569_1677del**	**FS**	**+**	**N**	**N**		**N**		**N**			**N**								
Δ(E3_E4,E8)	r.216_1314+1678_1810del	FS		N	N		N		N			N				+				
Δ(E3_E5)	r.216_1395del	FS			+										+					
Δ(E3,E5_E9)	r.216_364+1315_1903del	IF		N	N		N		N			N				+				
Δ(E3_E6)	r.216_1568del	IF	+	+	+	+		+		+	+			+	+	+	+	+	+	
Δ(E3_E6,E8)	r.216_1568+1678_1810del	FS		N	N		N	+	N		+	N								
Δ(E3_E7)	r.216_1677del	FS	+		+	+							+			+				
Δ(E3_E9)	r.216_1903del	FS	+		+	+										+		+		
Δ(E3q_E10)	r.361_2001del	IF													+					
Δ(E4)	r.365_1314del	FS	+	+	+	+	+	+	+	+	+	+	+	+	+	+	+	+	+	
**Δ(E4q137)**	**r.1178_1314del**	**FS**		**+**	**+**															
Δ(E4q408)	r.907_1314del	IF		+	+	+	+			+			+	+		+		+	+	
**Δ(E4int104)**	**r.718_821del**	**FS**		**+**	**+**															
**Δ(E4int695)**	**r.472_1166del**	**FS**		**+**	**+**	**+**							**+**							
Δ(E4,E6p)	r.365_?	?		N	N		N		N			N				+				
Δ(E4_E6)	r.365_1568del	FS		+	+	+							+		+					
Δ(E4q,E6q)	r.?_?	?		N	N		N		N			N				+				
Δ(E4_E7)	r.365_1677del	FS			+		+											+		
Δ(E4,E8_E9)	r.365_1314+1678_1903del	IF	+	N	N		N		N			N				+		+		
Δ(E4_E9)	r.365_1903del	IF	+		+	+				+				+		+	+	+	+	+
**IVS4-828**▼**38**	**r.1314_1315ins1315-828_1315-791**	**FS**		**+**	**+**	**+**				**+**			**+**							
**Δ(E5)**	**r.1315_1395del**	**IF**	**+**																	
**Δ(E5_E9)**	**r.1315_1903del**	**FS**			**+**															
**Δ(E5_E10)**	**r.1315_2001del**	**IF**				**+**				**+**			**+**							
▼**(I5q3)**	**r.1395_1396ins1395+1_1395+3**	**IF**		**+**	**+**	**+**				**+**			**+**							
**Δ(E6q2)**	**r.1567_1568del**	**FS**		**+**		**+**				**+**			**+**							
**IVS6+4684**▼**67**	**r.1568_1569ins1568+4684_1568+4752**	**FS**		**+**																
Δ(E7)	r.1569_1677del	FS	+	+	+	+	+			+			+							
**Δ(E7p35)**	**r.1569_1603del**	**FS**		**+**	**+**	**+**				**+**			**+**							
Δ(E7_E9)	r.1569_1903del	FS			+		+		+			+						+		
▼**(I7q4)**	**r.1677_1678ins1677+1_1677+4**	**FS**		**+**	**+**															
Δ(E8)	r.1678_1810del	FS		+	+	+	+			+			+					+		
Δ(E8_E9)	r.1678_1903del	FS		+	+		+											+		
**Δ(E9)**	**r.1811_1903del**	**IF**				**+**				**+**			**+**							
**Δ(E9p30)**	**r.1811_1840del**	**IF**		**+**	**+**	**+**				**+**			**+**							
▼**(I9q4)**	**r.1903_1904ins1903+1_1903+4**	**FS**		**+**	**+**	**+**				**+**										
IVS9+5946▼1015	r.1903_1904ins1903+5946_1904+6960	FS		+	+		+													
**IVS9-6318**▼**92**	**r.1903_1904ins1904-6318_1904-6227**	**FS**		**+**	**+**															
Δ(E10q4)	r.1998_2001del	FS			+	+	+			+			+							
IVS10+131▼46	r.2001_2002+131_2001+176	FS		+	+	+	+			+			+							

+, splicing events detected; N, splicing events not detectable from our analyses of short-read sequences; NS, nanopore sequencing; RP, RT-PCR; RS, whole RNA-seq; TRS, targeted RNA-seq. Novel splicing events identified in this study are shown in bold.

To compare the splicing data by assay used (nanopore sequencing/short-read RNA-seq *vs.* RT-PCR), we examined *BARD1* mRNA isoforms detected exclusively by one technology. In addition to the 12 splicing events detected by sequencing but not RT-PCR (listed above), 17 alternative splicing events were detected by RT-PCR but not characterized by long- or short-read RNA-seq assays (**Table 1**). Our short-read RNA-seq analyses were not able to characterize the complete exon structure for 12 of these 17 because they are compound events combining multiple non-contiguous splicing events. These results highlight a key limitation with assays that are unable to examine the entire transcript.

Many of the detected *BARD1* splicing events occurred at low levels (< 7% of the transcript pool) but were identified using newer and older technologies. The differences we have observed across different laboratories and published studies are possibly due to multiple factors including, different technologies with differing sensitivities, different sample types, different culture conditions, and different study cohort sizes. Such variability has also been observed between laboratories that used different cell processing and assay protocols for *BRCA1* and *BRCA2* isoform detection (Whiley et al., 2014). Events detected by only one laboratory or study are most likely due to reduced sensitivity of others methods to detect that particular event. However, we cannot exclude the possibility that some of these events maybe artifacts.

### Co-Occurring *BARD1* Splicing Events

Most RNA-seq technologies derive partial information about transcript structure due to targeting relatively short transcript sequences. Determining whether *BARD1* transcripts lead to abnormal and potentially deleterious proteins requires knowledge relating to the complete sequence structure of the coding isoforms. Using MinION (nanopore) sequencing of PCR amplified *BARD1* mRNA transcripts, we were able to sequence the full-length isoform along with 16 alternatively spliced isoforms accounting for 18 of the 62 individual splicing events (**Table 1**, **Supplementary Figure S2**). Two of the three novel isoforms found exclusively using this technology were out-of-frame [Δ(E2_E7) and Δ(E3_E4,E7)] and one was in-frame [Δ(E5)]. *BARD1* exon splicing events, such as Δ(E2_E4), Δ(E4), and Δ(E8), have been shown to co-occur independently in single transcripts as well as combined with other events to generate more complex isoforms.

Based on available data, the most complex *BARD1* transcript structures identified involved two alternative splicing events and was observed in 15 of the alternative transcripts (**Table 1**).

Although nanopore sequencing was conducted on PCR products generated from an LCL treated with an NMD inhibitor, we were not able to identify all junctions identified by short-read sequencing. This is likely a limitation of only sequencing amplicons derived from PCR assays using a single cell line. It is also important to note that we sequenced targeted amplicons which included exons 1 and 11, leaving the possibility that we excluded transcripts that do not contain these regions, such as Δ(E1–E4p) (**Table 1**). Analysis of truncated nanopore reads that do not contain exons 1 and 11 gave rise to several additional low confidence splicing events (**Supplementary Figure S2**). Results from the FLAIR bioinformatic analysis tool were presented in this study as this method has previously been shown to identify high-confidence spliced isoforms compared to other tools, such as Genomic Mapping and Alignment Program (GMAP) (Tang et al., 2018). Our re-analysis of nanopore sequence reads using the GMAP tool generated a list of 49 alternative *BARD1* transcripts including 11 splicing events that were not detected using the FLAIR analysis or by short-read RNA-seq and/or RT-PCR methods (**Supplementary Table S3**). These results suggest that the GMAP tool may be more sensitive than FLAIR, although the large number of novel splicing events detected also suggests a higher rate of false positive results, as previously reported (Tang et al., 2018).

### Relative Levels of *BARD1* Splicing

Relative expression levels of splicing events were determined using short-read RNA-seq analysis of LCLs cultured with and without an inhibitor of nonsense mediated decay (NMD). The most highly expressed alternative splicing events identified both in this study and that published by Davy et al. (Davy et al., 2017), using cells not treated with NMD inhibitors, produced out-of-frame transcripts and are shown in **Figure 1**. To assess the effect of NMD inhibitors on expression of splicing events we compared the percentage of sequenced junction reads corresponding to alternative splicing in treated cells with alternative splicing in non-treated cells. Results showed variable expression of splice junctions between the two groups (**Supplementary Figure S3**). For example, Δ(E4) is predicted to lead to the activation of a premature stop codon in exon 5 leading to NMD, however both laboratory 1 and 2 found that the percentage of junction reads for this event was greater in non-treated cells. Relatively low expression variability of *BARD1* splice junctions was observed between LCLs from laboratory 2 suggesting greater inter-laboratory variability than intra-laboratory variability (**Supplementary Figure S3**).

With the exception of Δ(E4), there was noticeable variability in the levels of splicing events detected across laboratories (**Figure 1**). None of these events exceeded 9% of the overlapping natural junctions in LCLs. However, Δ(E3) was expressed in breast tissue at ∼12% relative to the overlapping natural junctions. Since Δ(E3), and the other most highly expressed events, produce out-of-frame transcripts, this suggests that aberrant splicing is tolerated to at least this level. Interestingly, the level of Δ(E3) expression in colorectal tumor tissue has been shown to be associated with tumorigenesis and progression (Zhang et al., 2012), although it is unclear whether Δ(E3) expression levels in normal cells is associated with risk. Each exon deleted from the alternative transcripts overlapped a known functional domain of BARD1. The possible function of most isoforms identified to date remains unknown. However, several studies have shown that Δ(E2_E3) uses an alternative in-frame start codon and encodes a protein which has a proproliferative function despite losing the RING domain and therefore the ability to bind to BRCA1 (Li et al., 2007; Ryser et al., 2009; Zhang et al., 2012). Furthermore, evidence suggests that the in-frame isoforms Δ(E3_E6) and Δ(E3_E7) also have a role in cellular proliferation (Li et al., 2007). Apart from the Δ(E2_E3) isoform, there is little evidence to suggest that other out-of-frame transcripts [e.g., Δ(E3)] use an alternative open reading frame to encode functional proteins.

### *BARD1* Splicing and Interpretation for Variant Classification

Abou Tayoun et al. recently proposed a decision tree for interpreting the loss of function PVS1 ACMG/AMP criterion (Abou Tayoun et al., 2018). Regarding premature translation-termination codons (PTC-NMD variants) the guidelines suggest that they should be considered PVS1, unless located in an exon absent from biologically relevant transcript(s). For any PTC-NMD variants located in such exons, the PVS1 criterion is not applicable (N/A). This is a conservative rule introduced to cope with the possibility of rescue transcripts (i.e., alternatively spliced transcripts that skip the PTC-NMD variant providing haplo-sufficiency). Rescue transcripts overcoming the damaging effect of a PTC-NMD variant have been described for cancer predisposition genes such as *APC* (Nieuwenhuis and Vasen, 2007) and *BRCA1* (de la Hoya et al., 2016). However, we did not identify any candidate rescue transcript (no transcript other than the reference is predicted to code for functional RING, ARD, and BRCT domains) in our study. Therefore, we conclude that any PTC-NMD variant identified in *BARD1* should be considered PVS1. Regarding splice site (IVS ± 1,2) variants, ACMG/AMP guidelines are more complex, and splice site variants may be considered PVS1, PVS1_Strong, PVS1_moderate, or PVS1_not applicable depending on several factors, such as: 1) the predicted outcome of the splice alteration being in-frame or truncating; 2) the predicted impact on clinically functional domains of the protein; and 3) the presence of candidate rescue transcripts (**Supplementary Figure S4**). According to our analysis, *BARD1* variants located at consensus splice sites can be considered PVS1 (n = 9 sites), or PVS1_strong (n = 10 sites). Only variants located at the donor site of exon 5 should be considered PVS1_moderate (**Supplementary Table S4** and **Figure S4**). The presumed role of naturally occurring *BARD1* donor/acceptor shifts as predictors of cryptic site activation is based on a number of observations that we and others have made in other cancer susceptibility genes, including *PALB2, BRCA1*, and *BRCA2*. For example, *PALB2* c.48G > A (last nucleotide of exon 1) inactivates the donor site, leading to activation of a cryptic donor site to increasing levels of the naturally occurring Δ(E1q17) alternative splicing event (Lopez-Perolio et al., 2019). It is important to note that caution maybe warranted when assessing variants for potential associated donor/acceptor shifts in genes that have not been thoroughly investigated for alternative transcripts.

Thirty four variants located at *BARD1* canonical splice sites (gnomAD, ClinVar; accessed June 2019) were identified to assess their clinical significance using ACMG/AMP criteria adapted for *BARD1* as described in **Table 2**. In absence of *in vitro* studies, we conclude that these variants (all them absent or extremely rare in control populations, and therefore accounting for PM2) can be reported as likely pathogenic, with the exception of variants targeting the donor site of *BARD1* exon 5, for which we suggest a more conservative classification of uncertain significance (**Table 2**).

**Table 2:** Classification of canonical *BARD1* splice site variants using American College of Medical Genetics and Genomics and the Association for Molecular Pathology guidelines.

Exon	Splice site	Splice site variant (Ensembl/CinVar)	*In vitro* splicing data?	gnomAD (alleles)	ClinVar (review status)	PVS1	PM2^a^	Proposed ACMG/AMP classification^b^
1	158+1,+2	c.158+1G > T	No	0	Likely pathogenic (*)	PVS1	Yes	Likely pathogenic
2	159−1,−2	c.159−2A > G	No	1	Not reported	PVS1_strong	Yes	Likely pathogenic
2	159−1,−2	c.159−1G > T	No	0	Likely pathogenic (**)	PVS1_strong	Yes	Likely pathogenic
2	215+1,+2	215+2T > C	No	0	Likely pathogenic (*)	PVS1_strong	Yes	Likely pathogenic
3	216−1,−2	c.216−1G > A	No	0	Not reported	PVS1	Yes	Likely pathogenic
3	364+1,+2	Not reported	No	0	Not reported	PVS1	Yes	Likely pathogenic
4	365−1,−2	c.365−1G > T	No	0	Likely pathogenic (*)	PVS1_strong	Yes	Likely pathogenic
4	365−1,−2	c.365−2A > G	No	1	Not reported	PVS1_strong	Yes	Likely pathogenic
4	1314+1,+2	c.1314+1G > A	No	1	Likely pathogenic (**)	PVS1_strong	Yes	Likely pathogenic
5	1315−1,−2	c.1315−2A > G	Δ(E5) with RT-PCR E4–E6	1	Not reported	PVS1_strong	Yes	Likely pathogenic
5	1395+1,+2	c.1395+1dup	No	0	Likely pathogenic (**)	PVS1_moderate	Yes	Uncertain significance
6	1396−1,−2	Not reported	No	0	Not reported	PVS1	Yes	Likely pathogenic
6	1568+1,+2	c.1568+2T > C	No	0	Likely pathogenic (*)	PVS1	Yes	Likely pathogenic
7	1569−1,−2	Not reported	No	0	Not reported	PVS1	Yes	Likely pathogenic
7	1677+1,+2	c.1677+1G > C	No	0	Likely pathogenic (*)	PVS1	Yes	Likely pathogenic
7	1677+1,+2	c.1677+1G > A	No	1	Not reported	PVS1	Yes	Likely pathogenic
8	1678−1,−2	c.1678−1G > T	No	2	Not reported	PVS1	Yes	Likely pathogenic
8	1810+1,+2	c.1810+1G > A	No	0	Likely pathogenic (*)	PVS1	Yes	Likely pathogenic
8	1810+1,+2	c.1810+2T > G	No	0	Likely pathogenic (**)	PVS1	Yes	Likely pathogenic
9	1811−1,−2	c.1811−1G > A	No	0	Pathogenic (*)	PVS1_strong	Yes	Likely pathogenic
9	1903+1,+2	c.1903+1G > A	No	1	Not reported	PVS1_strong	Yes	Likely pathogenic
9	1903+1,+2	c.1903+1G > T	No	0	Likely pathogenic (*)	PVS1_strong	Yes	Likely pathogenic
10	1904−1,−2	c.1904−2A > T	No	0	Likely pathogenic (*)	PVS1_strong	Yes	Likely pathogenic
10	2001+1,+2	c.2001+1G > T	No	1	Uncertain significance (*)	PVS1_strong	Yes	Likely pathogenic
10	2001+1,+2	c.2001+1G > C	No	0	Likely pathogenic (**)	PVS1_strong	Yes	Likely pathogenic
10	2001+1,+2	c.2001+1G > A	No	0	Uncertain significance (*)	PVS1_strong	Yes	Likely pathogenic
10	2001+1,+2	c.2001+2T > C	No	1	Not reported	PVS1_strong	Yes	Likely pathogenic
11	2002−1,−2	c.2002−2A > G	No	1	Not reported	PVS1_strong	Yes	Likely pathogenic
11	2002−1,−2	c.2002−2A > C	No	0	Likely pathogenic(1); uncertain significance(1) (*)	1 PVS1_strong	2 Yes	3 Likely pathogenic
4 11	5 2002−1,−2	6 c.2002−2A > T	7 No	8 0	9 Likely pathogenic(1); uncertain significance(1) (*)	10 PVS1_strong	11 Yes	12 Likely pathogenic
13 11	14 2002−1,−2	15 c.2002−1G > A	16 No	17 1	18 Likely pathogenic (*)	19 PVS1_strong	20 Yes	21 Likely pathogenic
22 11	23 2002−1,−2	24 c.2002−1G > C	25 No	26 0	27 Likely pathogenic (*)	28 PVS1_strong	29 Yes	30 Likely pathogenic

^a^Absent.

^b^PM2 corresponds to the variant being absent from controls (or at extremely low frequency) as per ACMG/AMP guidelines (Richards et al., 2015). Gene specific ACMG/AMP guidelines have yet to be developed for BARD1, so it is possible that the use of PM2 and the weight of this criterion (e.g., moderate or supporting) may change in the future. *criteria provided/single submitter -or- conflicting interpretation, **multiple submitters/no conflicts.

In summary, we have conducted the most comprehensive assessment of *BARD1* mRNA splicing to date, and propose appropriate ACMG/AMP PVS1 evidence strengths to assist with classification of *BARD1* sequence variants in a modified version of the Abou Tayoun et al. (2018) decision tree. To our knowledge, we have conducted the first sequence analysis of whole *BARD1* mRNA transcript isoforms using nanopore sequencing, however further investigation of whole transcripts is required to account for all splicing events identified using other methods. This study did not identify *BARD1* candidate rescue transcripts, indicating that all premature translation-termination codons (PTC)_NMD variants can be assigned PVS1 at nominal strength. Moreover, donor and acceptor “consensus” dinucleotide variants (IVS+/− 1,2) can be considered PVS1 or PVS1_strong, with the possible exception of variants targeting the exon 5 donor site, which we recommend assigning PVS1_moderate.

## Data Availability Statement

The raw data supporting the conclusions of this manuscript will be made available by the authors, without undue reservation, to any qualified researcher.

## Ethics Statement

The studies involving human participants were reviewed and approved by University of Otago Human Ethics Committee (Health) - H14/131. The patients/participants provided their written informed consent to participate in this study.

## Author Contributions

MH conceived and supervised the study. All authors performed the experiments, conducted data analysis, and/or interpreted the experimental results. kConFab provided LCLs to Laboratories 1 and 4. LW wrote the manuscript. All authors made manuscript revisions

## Conflict of Interest

The authors declare that the research was conducted in the absence of any commercial or financial relationships that could be construed as a potential conflict of interest.
