# Supplementary material for: Comprehensive Assessment of BARD1 Messenger Ribonucleic Acid Splicing With Implications for Variant Classification
Source: Front Genet. 2019 Nov 19;10:1139. doi: 10.3389/fgene.2019.01139 (PMC6877745; doi:10.3389/fgene.2019.01139)
Supplement: Supplementary file 1 [file DataSheet_1.pdf]

Supplementary Methods: Splice site variant classification

To adapt the ACMG/AMP PVS1 decision tree (PMID: 30192042) to *BARD1* donor and acceptor "consensus" dinucleotide (IVS+/- 1,2) variants, we have used the following predictions and criteria:

Three regions critical to BARD1 function have been reported so far, the RING, ARD (Ankyrin Repeat Domain) and BRCT domains (PMID: 26738429). The RING domain spans residues 26 to 122 according to its solution structure (PMID: 11573085). The ARD spans residues 425_546 according to its crystal structure (PMID: 18480049). The BRCT domain spans residues 568_777 according to its crystal structure (PMID: 17550235).

We have not identified any *BARD1* transcript other than ENST00000260947 that is predicted to code for a protein with RING, ARD, and BRCT regions. Therefore, no candidate rescue transcript has been identified and, according to Tayoun et al (PMID: 30192042), all exons are present in a biologically relevant transcript.

After comprehensive review of the scientific literature, we have identified experimental splicing data only for one *BARD1* variant targeting a consensus splice site. The c.1315-2A>G variant (targeting exon 5 acceptor site) causes exon 5 skipping (PMID: 21344236). We have predicted that all putative variants targeting the same acceptor site will cause exon 5 skipping.

Lacking experimental data for the remaining donor/acceptor sites, we have performed predictions as follows: i) all variants are predicted to cause exon skipping, ii) variants are predicted as well to cause multi-exon skipping if the multi-cassette event has been observed in control samples (present study), iii) variants are predicted as well to cause activation of a cryptic site if the corresponding donor/acceptor shift has been observed in control samples (present study).

As indicated by Tayoun et al (PMID: 30192042), if multiple predictions can be made for impact of a single variant, the lowest strength scenario is selected for PVS1 decision tree. The strength scenario has been decided as follows (lowest to higher): i) in-frame not targeting regions critical for protein function, ii) in-frame targeting regions critical representing <10% length of protein, iii) in-frame targeting regions critical representing >10% length of protein, iv) out-of-frame.

All splice sites with in-frame prediction targeting the RING, ARD, and BRCT regions have been considered PVS1_strong, the only exception being the exon 5 donor site (c.1315+1,+2). Variants targeting this site are predicted to cause exon 5 skipping, and/or exon 5 to 9 skipping, and/or exon 5 to 10 skipping, and/or use of a cryptic donor site in intron 5. The latter is the lowest strength scenario, as is predicted to insert 3 nucleotides in the coding sequence. The predicted consequence at the protein level will be p.Leu465_His466ins1, with the precise residue to be inserted (Leu, Ile, Ala, Glu, or Gly), depending on the actual variant substitution. Despite targeting the ARD domain, it is far from obvious that insertion of one residue in this region will be damaging, so that we have decided to be conservative and annotate c.1315+1,+2 variants as PVS1_Moderate.
