# Supplementary figures and images for: Comprehensive Assessment of BARD1 Messenger Ribonucleic Acid Splicing With Implications for Variant Classification
Source: Front Genet. 2019 Nov 19;10:1139. doi: 10.3389/fgene.2019.01139 (PMC6877745; doi:10.3389/fgene.2019.01139)

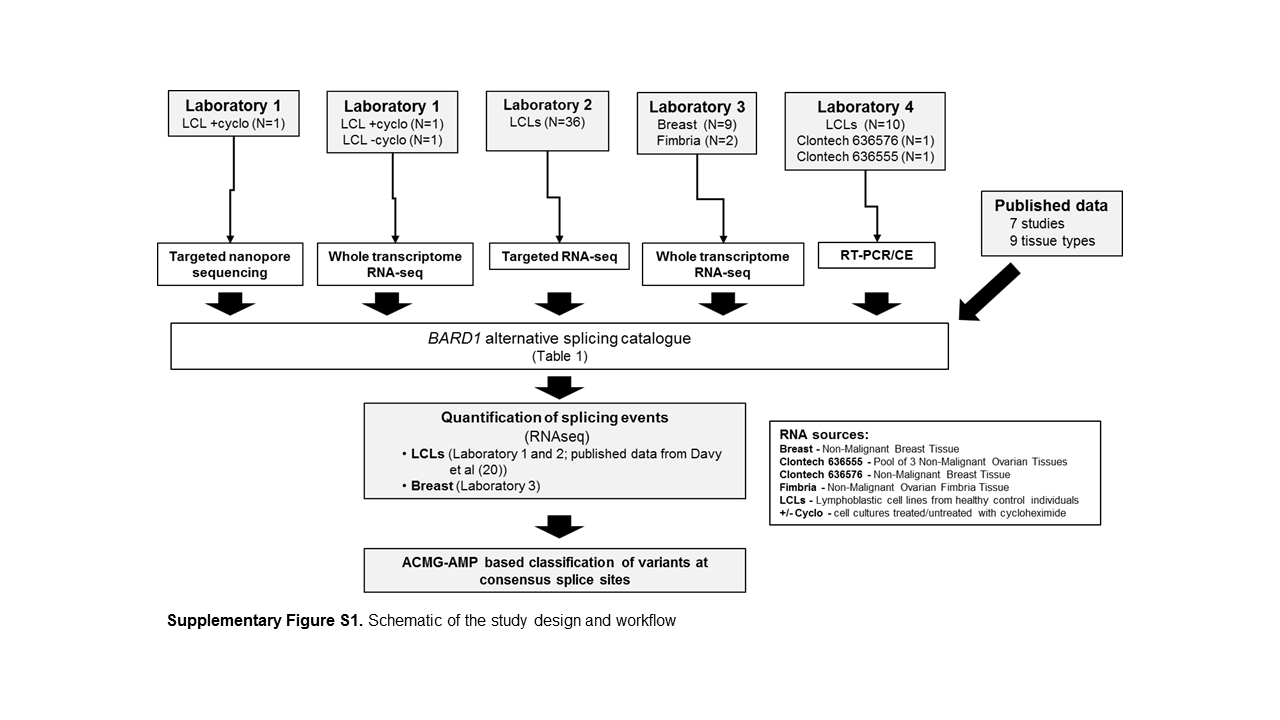

Supplement: Supplementary file 2 [file Image_1.tif]

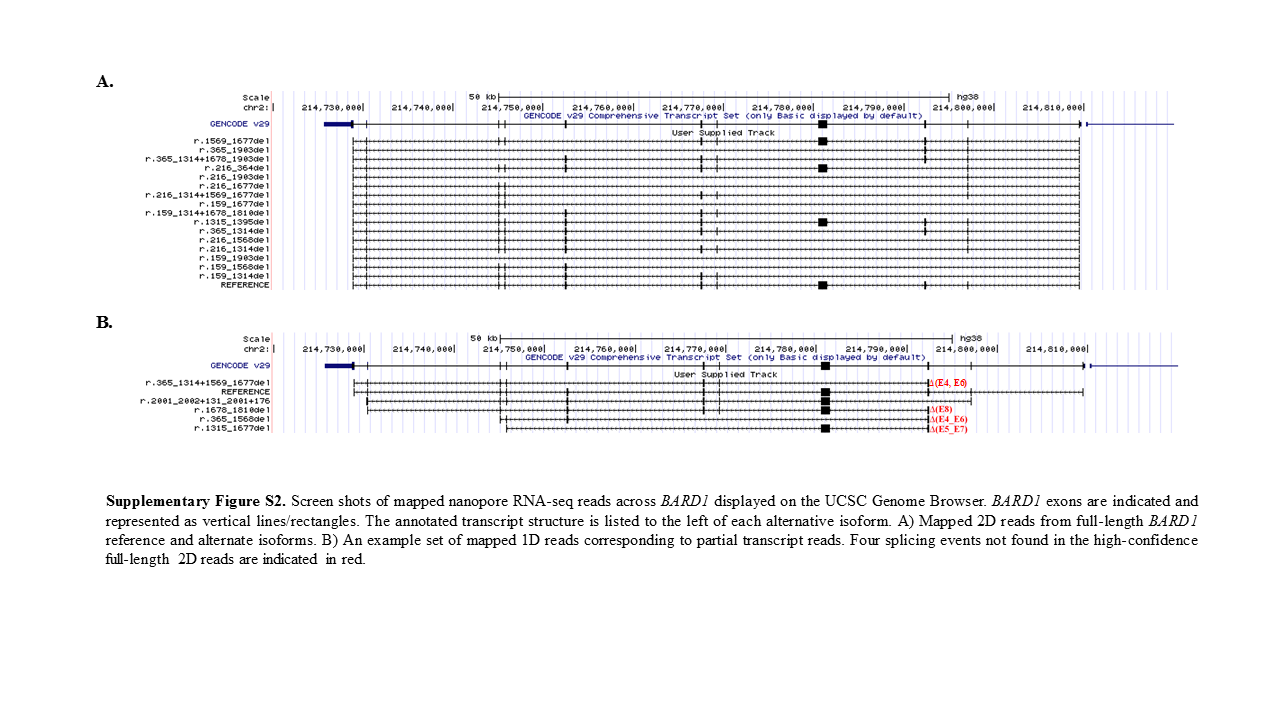

Supplement: Supplementary file 3 [file Image_2.tif]

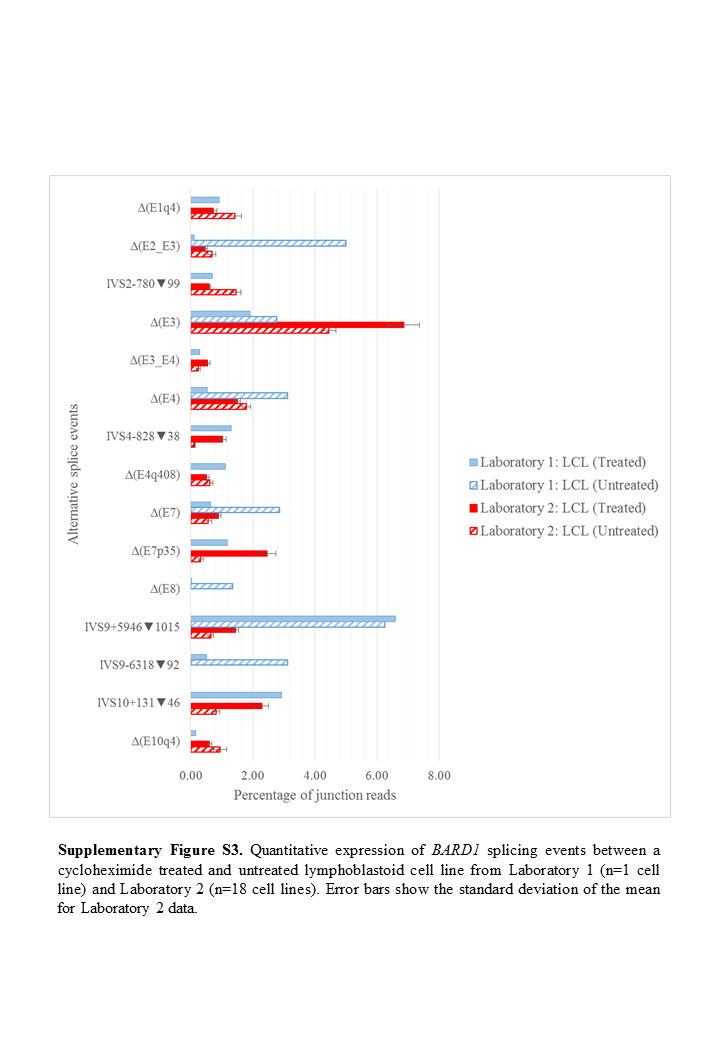

Supplement: Supplementary file 4 [file Image_3.tif]

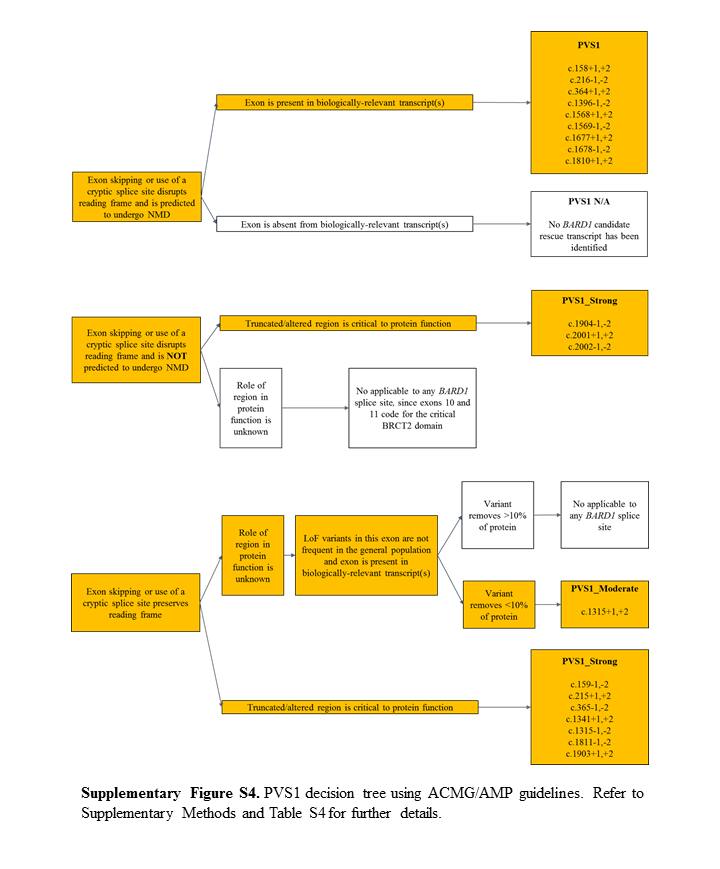

Supplement: Supplementary file 5 [file Image_4.tif]
